# Shockwaves prevent from heart failure after acute myocardial ischaemia *via* RNA/protein complexes

**DOI:** 10.1111/jcmm.13021

**Published:** 2016-12-20

**Authors:** Can Tepeköylü, Uwe Primessnig, Leo Pölzl, Michael Graber, Daniela Lobenwein, Felix Nägele, Elke Kirchmair, Elisabeth Pechriggl, Michael Grimm, Johannes Holfeld

**Affiliations:** ^1^ Department for Cardiac SurgeryMedical University of InnsbruckInnsbruckAustria; ^2^ Department of CardiologyCharité University BerlinBerlinGermany; ^3^ Division of Clinical and Functional AnatomyDepartment of Anatomy, Histology and EmbryologyInnsbruck Medical UniversityInnsbruckAustria

**Keywords:** shock wave therapy, myocardial regeneration, inflammation, myocardial infarction

## Abstract

Shock wave treatment (SWT) was shown to induce regeneration of ischaemic myocardium *via* Toll‐like receptor 3 (TLR3). The antimicrobial peptide LL37 gets released by mechanical stress and is known to form complexes with nucleic acids thus activating Toll‐like receptors. We suggested that SWT in the acute setting prevents from the development of heart failure *via* RNA/protein release. Myocardial infarction in mice was induced followed by subsequent SWT. Heart function was assessed 4 weeks later *via* transthoracic echocardiography and pressure–volume measurements. Human umbilical vein endothelial cells (HUVECs) were treated with SWT in the presence of RNase and proteinase and analysed for proliferation, tube formation and LL37 expression. RNA release and uptake after SWT was evaluated. We found significantly improved cardiac function after SWT. SWT resulted in significantly higher numbers of capillaries and arterioles and less left ventricular fibrosis. Supernatants of treated cells activated TLR3 reporter cells. Analysis of the supernatant revealed increased RNA levels. The effect could not be abolished by pre‐treatment of the supernatant with RNase, but only by a sequential digestion with proteinase and RNase hinting strongly towards the involvement of RNA/protein complexes. Indeed, LL37 expression as well as cellular RNA uptake were significantly increased after SWT. We show for the first time that SWT prevents from left ventricular remodelling and cardiac dysfunction *via* RNA/protein complex release and subsequent induction of angiogenesis. It might therefore develop a potent regenerative treatment alternative for ischaemic heart disease.

## Introduction

Myocardial ischaemia with consequent loss of cardiomyocytes can cause left ventricular remodelling including development of fibrotic scar tissue, thinning of the myocardium and decreased systolic function resulting in heart failure. Affected patients suffer from dyspnoea, fatigue and oedema, show high morbidity and mortality and represent a major socio‐economic health burden for Western countries 1, 2. Current treatment strategies provide symptomatic relief 3. However, no currently available treatment option is able to avoid ventricular remodelling with subsequent development of heart failure after myocardial injury.

Shock waves are mechanical pressure waves which have been used in medicine for kidney stone lithotripsy for more than 30 years 4. In lower energies, they were shown to induce tissue regeneration *via* induction of angiogenesis 5, mobilization of progenitor cells 6, 7 and alteration of inflammatory response 8. Therefore, they have been used successfully in clinical routine for regenerative purposes, for example in bone non‐unions 9, soft tissue wounds 10 and burn injuries 11. Cardiac SWT application showed induction of angiogenesis 5, improved function and caused symptom relief in patients suffering from chronic ischaemic heart disease 12. The exact working mechanism remains not fully understood.

We could describe only recently that the regenerative effects of SWT are mediated *via* RNA release and subsequent TLR3 activation. SW effects were missing completely in TLR3^−/−^ animals 13. However, extracellular self‐RNA is degraded immediately to avoid immunogenic response 14, 15. Therefore, it remains unknown how extracellular RNA released upon SWT (1) escapes from degradation and (2) can activate intracellular TLR3.

Extracellular RNA mainly occurs in protein‐complexed forms, and thus escapes degradation 16. Thereby, the cationic antimicrobial peptide LL37 has been described to be released upon mechanical stress, to form complexes with ribonucleic acids, to pass the cellular membrane and to subsequently activate intracellular TLRs 15. We therefore suggested that SWT causes release of LL37. The peptide forms complexes with extracellular RNA, passes the cell membrane and activates TLR3.

In this study, we aimed to investigate whether (1) SWT in the acute setting of myocardial infarction prevents from the development of ischaemic heart failure, and (2) SWT effects are indeed mediated *via* LL37/RNA complexes.

## Materials and methods

### Animal experiments

The experiments were approved by the institutional animal care and use committee at Innsbruck Medical University and by the Austrian ministry of science. The investigation conformed to the ‘Guide for the Care and Use of Laboratory Animals’ published by the U.S. National Institutes of Health (NIH Publication No. 85‐23, revised 1996; available from: www.nap.edu/catalog/5140.html). Male, 12‐ to 14‐week old C57/BL6 mice (Charles River, Sulzfeld, Germany) weighing 25–30 g were randomly divided into two groups (shock wave therapy group = SWT and untreated control group = CTR, *n* = 6). SWT animals received shock wave therapy directly after LAD ligation. CTR animals were left untreated. The animals were killed 4 weeks after therapy for harvesting of the heart.

### Induction of myocardial infarction

Myocardial infarction was induced as it is the standard model for the induction of ischaemic cardiomyopathy. It was performed as described previously 17. Briefly, animals were anesthetized by an intraperitoneal injection of ketamine hydrochloride (Graeub, Bern, Switzerland; 80 mg/kg bodyweight) and xylazine hydrochloride (aniMedica, Senden, Germany; 5 mg/kg bodyweight). A left lateral thoracotomy was performed in the fourth intercostal space for exposure of the heart. The left anterior descending artery (LAD) was ligated at the level of the pulmonary artery using 7‐0 polypropylene sutures (Ethicon, Somerville, NJ, USA).

### SWT

Animals were anesthetized for SWT as described above. Commercially available ultrasound gel was used for coupling. The commercially available Orthogold 180 with the hand‐held applicator CG050‐P (TRT LLC, Tissue Regeneration Technologies, Woodstock, GA, USA), which was specifically designed for cardiac shock wave therapy, was used for the treatment. Three‐hundred impulses were delivered to the heart through the thorax aiming at the ischaemic area at an energy flux density of 0.38 mJ/mm^2^ at a frequency of 5 Hz. At these energy levels, no adverse effects could be observed. However, for all *in vitro* experiments, 250 impulses with an energy flux density of 0.08 mJ/mm^2^ at a frequency of 3 Hz were used. 17 The rationale of the treatment parameters is our experience from previous studies 18.

### Haemodynamic pressure–volume measurements

Haemodynamic pressure–volume analysis and transthoracic echocardiography were performed, as the combination of these methods allows exact measurement of cardiac function. Invasive haemodynamic measurements and the analysis of pressure–volume (PV) loops were performed as terminal procedure according to established protocols described previously 18 using a pressure‐volume conductance system (MPVS Ultra; Millar Instruments, Houston, TX, USA) connected to the PowerLab 8/35 data acquisition system, United Kingdom, and analysed using LabChart7pro software system (AdInstruments, Colorado Srpings, CO, USA), United Kingdom. Mice were anaesthetized (3% isoflurane and 96–97% O_2_), intubated and ventilated by a small rodent ventilator. The animals were placed on a temperature‐controlled heating platform, and the core temperature was maintained at 37.5°C. Anaesthesia was reduced and kept at 1.5% isoflurane and 98.5% O_2_. A polyethylene catheter was inserted into the right external jugular vein for saline calibration (10% NaCl) at the end of the experiment to calculate the parallel volume. The 1.4‐F PV conductance catheter (SPR‐839; Millar) was inserted into the right carotid artery and advanced into the ascending aorta. Aortic (systemic) blood pressure and heart rate were recorded after stabilization for 5 min. Then, the catheter was advanced into the LV under pressure control through the aortic valve. Parameters of systolic and diastolic function, including LV end‐diastolic pressure (LVEDP), LV end‐systolic volume (LVESV), LV end‐diastolic volume (LVEDV), ejection fraction (EF), maximal slope of LV systolic pressure increment (d*P*/d*t* max) and maximal slope of diastolic pressure decrement (d*P*/d*t* min), were measured and calculated according to standard formulas. After baseline measurements, transient occlusion of the inferior vena cava was performed and used to derive end‐systolic (Ees) and end‐diastolic pressure–volume relationships (EDPVR) as load‐independent measures of cardiac contractility and relaxation, respectively. Heart rate (HR) was separately measured *via* surface ECG electrodes.

### Echocardiography

Transthoracic echocardiography was performed using standardized protocols for the assessment of heart function and morphometry as described previously 19, 20. Briefly, lightly anaesthetized mice (0.5% isoflurane and 99.5% O_2_) were placed on a temperature‐controlled warming pad (kept at 37.5°C) and imaged in the supine position using a high‐resolution micro‐imaging system equipped with a 30‐MHz linear array transducer (Vevo770TM Imaging System; VisualSonics Inc., Toronto, Ontario, Canada), respectively. Standard 2D‐ and M‐mode tracings of the left ventricle (LV; long axis and short axis at papillary muscle level) were recorded, and ejection fraction was averaged from three consecutive cardiac cycles under stable conditions. M‐Mode pictures were recorded in the mid‐papillary portion as standard in the Teichholz measuring technique. The investigator was blinded to the treatment.

### Cell culture and *in vitro* assays

Cell culture and *in vitro* assays were performed to investigate underlying mechanisms of SW‐induced angiogenesis. For this purpose, human umbilical vein endothelial cells (HUVECs) were cultured as described previously 21. Permission was given from the ethics committee of Innsbruck Medical University (No. UN4435). Cells were used until passage 5. All *in vitro* experiments were performed in triplicates.

In all experiments, 250 impulses were applied to the SWT group at an energy flux density of 0.08 mJ/mm^2^ and a frequency of 3 Hz. These parameters were chosen due to our experience from previous experiments 21, 22.

For assessment of proliferation, a BrdU assay (Roche, Rotkreuz, Switzerland) was performed as recommended by the manufacturer. Tube formation assay was performed as described previously 23. Tube formation was analysed using the ImageJ plugin Angiogenesis Analyzer as described previously 17. Both assays are recognized assays for the assessment of *in vitro* angiogenesis. For reporter cell assays, a HEK reporter cell line (TLR3/ISRE LUCPorter; Imgenex, San Diego, CA, USA) was purchased and used as suggested by the manufacturer. Cells were treated with SWT as described above. 24 hrs after treatment, cells were washed with PBS and luminescence was analysed using a Luciferase Assay System (Promega, Fitchburg, WI, USA).

### RNA content and uptake

RNA content was measured as described previously, as it represents a feasible technique for RNA content measurement 13. Rhodamine labelled polyinosinic:polycytidylic acid (poly (I:C)) (Invivogen, San Diego, CA, USA) was added to HUVEC supernatant prior to SWT treatment. Twenty‐four hours after, SWT cells were washed with PBS, cell membranes were stained using wheat germ agglutinin (WGA; Life Technologies, Carlsbad, CA, USA) and nuclei were visualized using DAPI. Rhodamine fluorescence was quantified using ImageJ. This method has proven useful for the investigation of RNA uptake 15. Results are depicted as positive area.

### Western blot

Western blot for protein expression was performed as described previously 13. The blots were probed with LL‐37 antibody (Abcam, Cambridge, UK).

### Immunofluorescence and histological staining

Immunofluorescence staining was performed as described previously to analyse proliferation and number of vessels 13. Histological sections of the heart were incubated with monoclonal rat anti‐CD31 (nova, Hamburg, Germany) rabbit polyclonal anti‐alpha smooth muscle actin antibodies (Abcam), rabbit anti‐Ki 67 or rabbit anti‐LL37 over night at 4°C. Masson–Goldner trichrome staining was performed as suggested by the manufacturer (Carl Roth GmbH, Karlsruhe, Germany) to quantify post‐infarctional fibrosis. The area of fibrosis and total area of the left free ventricle were measured using ImageJ (NIH, Bethesda, MD, USA). Values are shown as the ratio of fibrotic area to total left ventricular free wall as described previously 24. Five areas per sample were analysed. Three random pictures per sample were analysed. Sections were examined with a Zeiss Axioplan 2 (Zeiss, Oberkochen, Germany) and a Leica SP5 confocal microscope (Leica, Wetzlar, Germany). Images were analysed using ImageJ software (National Institutes of Health) and processed with Adobe Photoshop CS5.1 for Mac (Adobe Systems Inc., San Jose, CA, USA). Analyses were performed by a single blinded researcher.

### ELISA

A LL‐37 ELISA kit was used to analyse LL37 release to the supernatant (Hycult Biotech, Uden, the Netherlands). The assay was performed as recommended by the manufacturer.

### Statistical analysis

All results are expressed as mean + S.E.M. Statistical comparisons between two groups were performed by Student's *t*‐test or Mann–Whitney *U*‐test as appropriate. Multiple groups were analysed by two‐way anova followed by Bonferroni's multiple comparison test to determine statistical significance. Probability values <0.05 were considered statistically significant. All experiments were repeated at least in triplicate.

## Results

### SWT prevents from heart failure after acute myocardial infarction

**Figure 1:**
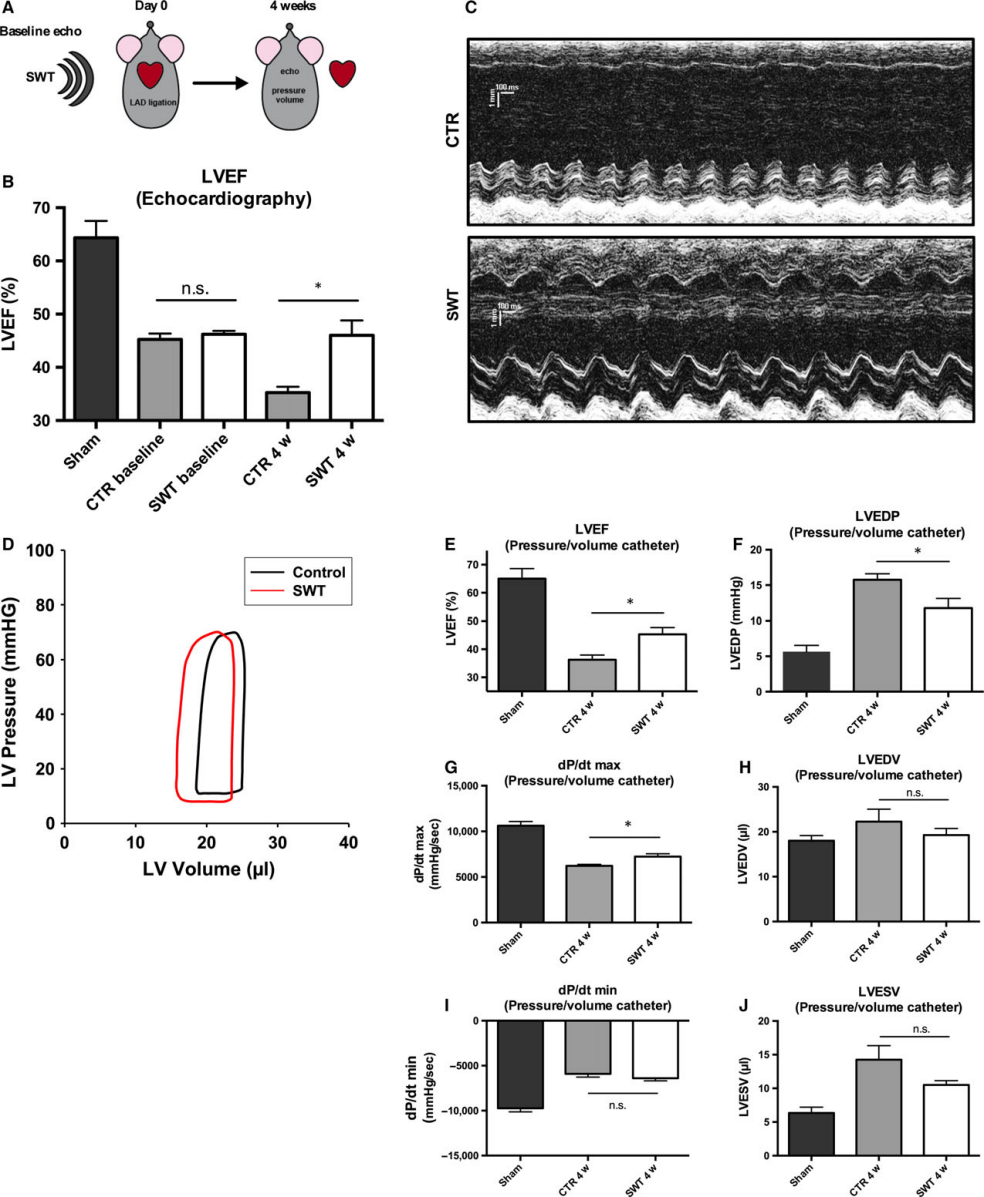
Shock wave treatment (SWT) prevents from heart failure after acute myocardial infarction. (**A**) Myocardial infarction was induced *via* LAD ligation. Subsequently, baseline measurement was preformed *via* echocardiography. Animals were treated with SWT immediately thereafter. Four weeks later, cardiac function was assessed *via* transthoracic echocardiography and pressure–volume measurement. (**B**) Baseline echo showed no difference between the groups before treatment. However, treated animals showed significantly improved left ventricular ejection fraction (LVEF) 4 weeks after treatment compared with untreated controls. (**C**) Representative M‐mode images from the transthoracic echocardiographies. The left ventricular anterior wall shows improved contractility after SWT compared to untreated controls. (**D**) Cardiac function was assessed in more detail using pressure–volume measurements. (**E**) Improved LVEF after SWT could be confirmed in pressure–volume measurements. In addition, treated animals showed decreased left ventricular end‐diastolic pressure (LVEDP;** F**), higher d*P*/d*t* max (**G**), decreased left ventricular end‐diastolic volume (LVEDV;** H**), d*P*/d*t* min. (**I**) and left ventricular end‐systolic pressure (LVESV;** J**). Overall, diastolic and systolic function as well as myocardial contractility showed improvement after SWT. *n* = 6, *P < 0.05.

The design of the *in vivo* experiment is depicted in Figure 1(A). Baseline echocardiography showed no difference between the groups before treatment (ejection fraction (EF) in %: CTR 45.25 ± 1.11 *versus* SWT 46.2 ± 0.66, *P* = 0.47; Fig. 1B). Four weeks after induction of myocardial infarction and subsequent shockwave treatment, transthoracic echocardiography showed significantly improved ejection fraction in treated animals compared with untreated control animals (LVEF in %: CTR 35.25 ± 1.11 *versus* SWT 46 ± 2.83, *P* = 0.0122; Fig. 1B and C). This finding could be confirmed by haemodynamic pressure‐volume measurement (LVEF in %: CTR 36.25 ± 1.70 *versus* SWT 45.25 ± 2.43, *P* = 0.0029; Fig. 1D and E). In addition, a decreased left ventricular end‐diastolic pressure (LVEDP) following SWT was found (Fig. 1F; mmHg: CTR 15.75 ± 0.85 *versus* SWT11.75 ± 1.38, *P* = 0.0485). Myocardial contractility was increased in treated animals (Fig. 1G and I; d*P*/d*t* max in mmHg/sec.: CTR 6222 ± 157.7 *versus* SWT 7236 ± 313.5, *P* = 0.0276; d*P*/d*t* min in mmHg/sec.: CTR −5906 ± 368 *versus* SWT −6396 ± 289, *P* = 0.3354). Moreover, left ventricular end‐diastolic volume (Fig. 1H; LVEDV) was decreased and left ventricular end‐systolic volume (LVESV) was increased after SWT, however, not significantly (Fig. 1J; LVEDV in μl: CTR 22.25 ± 2.78 *versus* SWT 19.256 ± 1.49, *P* = 0.3785; LVESV in μl: CTR 14.25 ± 2.10 *versus* SWT 10.5 ± 0.65, *P* = 0.1382).

### SWT induces angiogenesis and avoids myocardial scar formation

**Figure 2:**
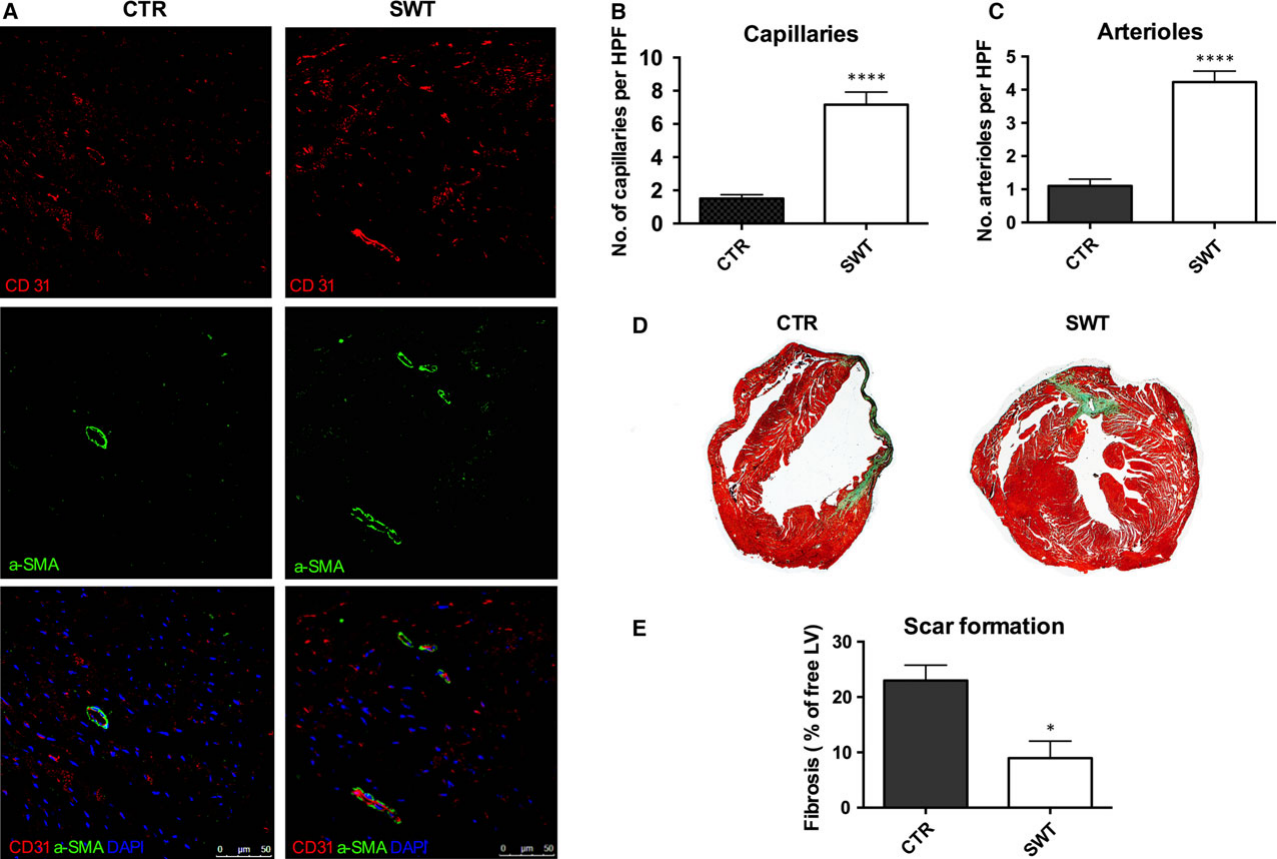
Shock wave treatment (SWT) induces angiogenesis and avoids myocardial scar formation. (**A**) Representative images of myocardium analysed for capillaries (CD31) and arterioles (aSMA) *via* immunofluorescence staining. (**B**) SWT resulted in significantly increased numbers of capillaries in the myocardium. (**C**) In addition, animals showed an increased amount of arterioles after treatment. (**D**) Masson's trichrome staining was used to quantify the amount of fibrosis of the left ventricle. (**E**) We found a significantly decreased amount of fibrosis in treated animals. *n* = 6, *P < 0.05, ****P < 0.0001.

In a next step, we aimed to clarify whether SWT resulted in angiogenesis and reduction in the fibrotic scar (Fig. 2A). Treated hearts showed significantly increased numbers of capillaries (Fig. 2B; capillaries per HPF: CTR 1.5 ± 0.23 *versus* SWT 7.17 ± 0.76, *P* < 0.0001) and arterioles (Fig. 2C; arterioles per HPF: CTR: 1.1 ± 0.21 *versus* SWT 4.23 ± 0.32, *P* < 0.0001). In addition, we found significantly decreased amounts of fibrosis after SWT (% of free LV: CTR ± 2.76 *versus* SWT 8.97 ± 3.08, *P* = 0.0132; Fig. 2D and E).

### SWT causes RNA release and promotes cellular uptake

**Figure 3:**
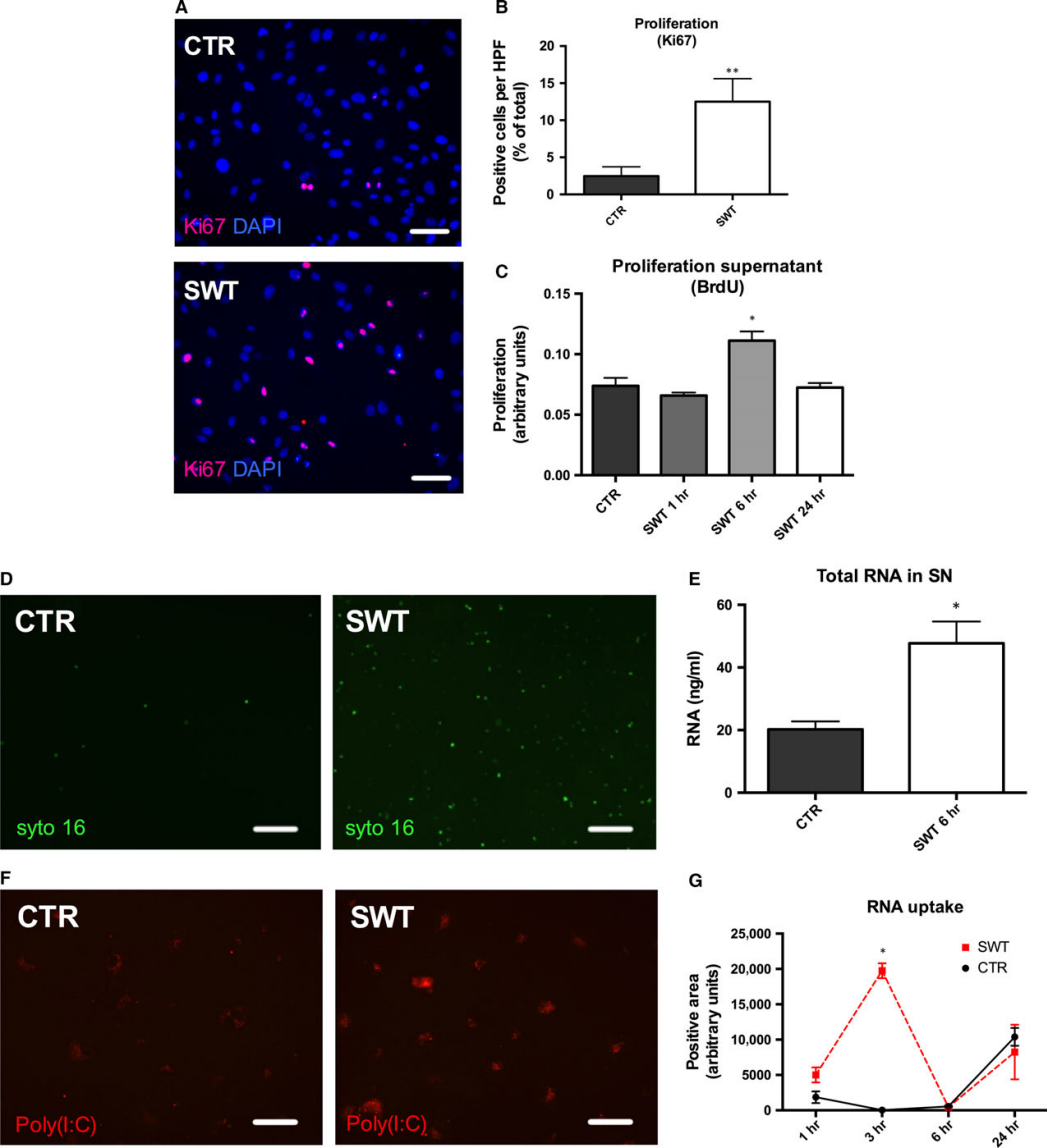
Shock wave treatment (SWT) causes RNA release and promotes cellular uptake. (**A**) Representative images of HUVECs treated with SWT and analysed for proliferation using Ki67 staining. (pink = Ki67, blue = DAPI, scale bar = 100 μm; **B**) SWT resulted in proliferation of HUVECs compared to untreated control cells. (**C**) Pre‐conditioned supernatant obtained from SW‐treated cells showed the same effect as direct SW application. (**D**) HUVECs were treated with SWT and analysed for RNA content (green = Syto16, scale bar = 100 μm). (**E**) We found significantly increased amounts of RNA in the supernatant of SW‐treated cells compared with untreated control supernatant. (**F**) Pre‐marked RNA was added to HUVECs prior to SWT. Subsequently, SWT was applied and RNA uptake quantified (red = poly(I:C), scale bar = 100 μm). (**G**) Cellular RNA uptake was significantly increased after SWT. Experiments were performed at least in triplicates and repeated at least twice. *P < 0.05, **P < 0.01.

Endothelial cells were treated with SWT and analysed for proliferation (Fig. 3A). We found increased proliferation after treatment (Fig. 3B; % of Ki67‐positive cells: CTR 2.46 ± 1.25 *versus* SWT 12.51 ± 3.10, *P* = 0.0047). Next, we aimed to clarify whether the observed effect was due to a factor that was released into the supernatant. Treatment with pre‐conditioned medium indeed resulted in higher proliferation rates (compared to cells treated with unconditioned medium; Fig. 3C; arbitrary units: CTR 0.07 ± 0.01 *versus* SWT 1 hr 0.07 ± 0.003, *P* = 0.28; SWT 6 hrs 0.11 ± 0.01, *P* = 0.0019 *versus* CTR; SWT 24 hrs 0.07 ± 0.004, *P* = 0.86 *versus* CTR). Next, we analysed the RNA content in treated supernatants. We found a significantly increased amount of total RNA in supernatants after SWT compared to untreated controls (ng/ml: CTR 20.21 ± 2.56 *versus* SWT 47.7 ± 6.97, *P* = 0.0023; Fig. 3D and E). Addition of pre‐marked RNA to cell culture supernatants prior to SWT showed an increased uptake 3 hrs after treatment (Fig. 3F and G; arbitrary units: 1 hr: CTR 1855 ± 824.7 *versus* SWT 5007 ± 1061, *P* = 0.0625; 3 hrs: CTR 31.67 ± 28.17 *versus* SWT 19,757 ± 1054, *P* = 0.0001; 6 hrs: CTR 545.7 ± 81.62 *versus* SWT 453.3 ± 44.67, *P* = 0.3722; 24 hrs: CTR 10,387 ± 1261 *versus* SWT 8249 ± 3862, *P* = 0.6267).

### RNA/protein complexes mediate SW effect

**Figure 4:**
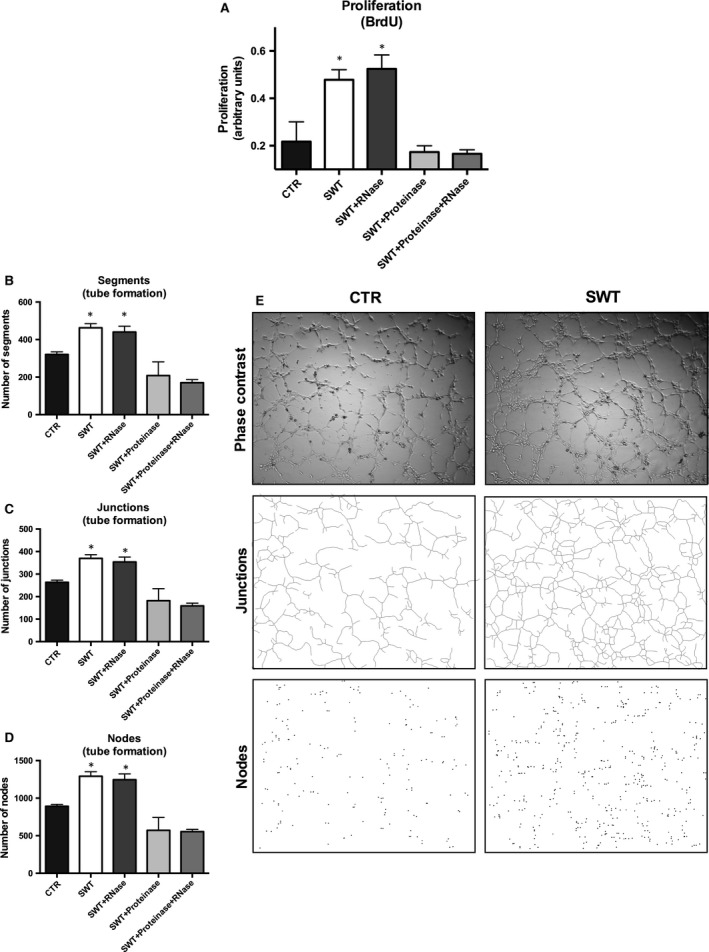
RNA/protein complexes mediate SW effect. (**A**) SWT enhanced proliferation in HUVECs. The effect could not be abolished *via* RNase pre‐treatment, but only with additional proteinase treatment. (**B**) SWT resulted in increased segment formation, formation of junctions (**C**) and enhanced node formation (**D**) in a tube formation assay. Again, the effect could not be abolished by RNase treatment, but only with proteinase addition. (**E**) We found significantly more tubes after SWT of HUVECs. Experiments were performed at least in triplicates and repeated at least twice. *P < 0.05.

Next, we added either RNase or proteinase and a combination of both to the endothelial cells prior to SWT and again analysed for proliferation. RNase alone did not abolish the SWT effect (arbitrary units: CTR 0.22 ± 0.08, SWT 0.48 ± 0.04, *P* = 0.02 *versus* CTR; SWT + RNase 0.52 ± 0.06, *P* = 0.02 *versus* CTR). However, SW effects were abolished in the presence of proteinase (SWT + Proteinase 0.17 ± 0.03, *P* = 0.5312 *versus* CTR; Fig. 4A). The results were confirmed in a tube formation assay showing increased number of segments (Fig. 4B; segments per HPF: CTR 320.8 ± 13.78 *versus* SWT 462.5 ± 22.26, *P* = 0.0015), junctions (Fig. 4C; junctions per HPF: CTR 263.5 ± 9.82 *versus* SWT 369.7 ± 16.4, *P* = 0.0013) and nodes (Fig. 4D; nodes per HPF: CTR 891.3 ± 25.96 *versus* SWT 1292 ± 60.11, *P* = 0.0029) after SWT. The effects could not be reversed by addition of RNase, only treatment with additional proteinase resulted in abolished SW effects (segments per HPF: SWT+RNase: 440 ± 31.42, *P* = 0.0186 *versus* CTR; SWT+Proteinase: 208 ± 73.37, *P* = 0.136 *versus* CTR; SWT+Proteinase+RNase: 170 ± 16.56, *P* = 0.0009 *versus* CTR; junctions per HPF: SWT+RNase: 354.2 ± 21.29, *P* = 0.0113; SWT+Proteinase: 182 ± 53.03, *P* = 0.1357 *versus* CTR; SWT+Prot+RNase: 159 ± 12.11, *P* = 0.0005 *versus* CTR; nodes per HPF: SWT+RNase 1246 ± 77.42, *P* = 0.0175 *versus* CTR; SWT+Proteinase 572 ± 170.9, *P* = 0.1771 *versus* CTR; SWT+Proteinase+RNase 554.8 ± 29.93, *P* = 0.0005 *versus* CTR).

### Increased LL37 expression after SWT

**Figure 5:**
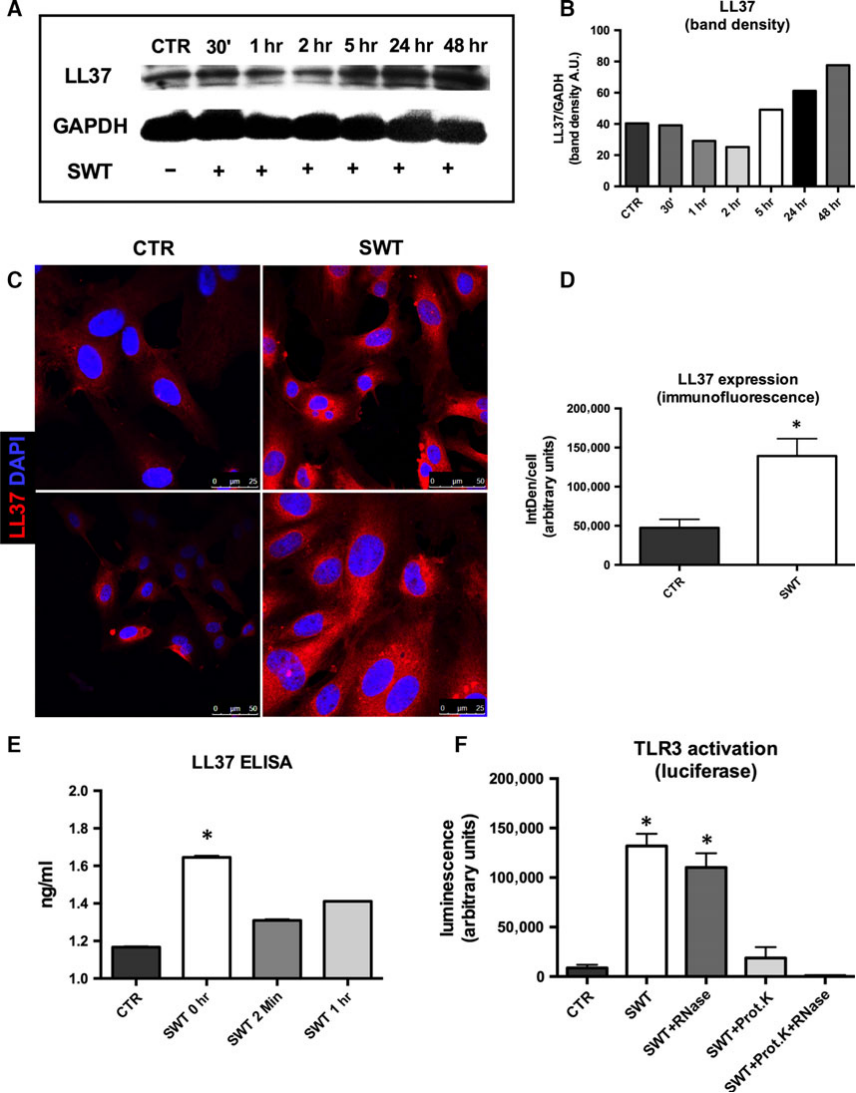
Increased LL37 expression after SWT. (**A**) Western blot analysis was performed to assess LL37 expression. (**B**) SW‐treated cells showed significantly increased LL37 expression after SWT compared to untreated controls. (**C**) Immunofluorescence staining for LL37 of HUVECs with and without treatment was performed. (**D**) After SWT, LL37 expression was significantly increased. (**E**) To assess LL37 release upon SWT, a LL37 ELISA of pre‐conditioned supernatants was performed. We found significantly increased amount of LL37 1 hr after SWT. (**F**) Finally, TLR3 activation after SWT was measured using a reporter cell assay. TLR3 activation was significantly increased after SWT. The effect was not abolished upon RNase addition, but only with addition of proteinase. Experiments were performed at least in triplicates and repeated at least twice. *P < 0.05.

LL37 expression was increased after SWT as shown by Western blot analysis (Fig. 5A and B; arbitrary units: CTR 40.35, SWT 30’ 39.13, SWT 1 hr 29.15, SWT 2 hrs 25.25, SWT 5 hrs 49.14, SWT 24 hrs 61.18, SWT 48 hrs 77.59) as well as immunofluorescence analysis (Fig. 5C and D; arbitrary units: CTR 47,496 ± 10,690 *versus* SWT 139,243 ± 22,250, *P* = 0.0054). In addition, LL37 content in the supernatant was also increased after SWT (Fig. 5E; ng/ml: CTR 1.17 ± 0.0005 *versus* SWT 0 hr 1.65 ± 0.008, *P* < 0.0001, SWT 2 min. 1.30 ± 0.006, *P* < 0.0001 *versus* CTR; SWT 1 hr 1.41 ± 0.002, *P* < 0.0001 *versus* CTR; SWT 6 hrs 1.52 ± 0.008, *P* < 0.0001 *versus* CTR; SWT 24 hrs 1.32 ± 0.005, *P* < 0.0001).

Finally, we transferred pre‐treated supernatant onto TLR3 reporter cells. Supernatant from SW‐treated cells caused TLR3 activation (arbitrary units: CTR 8728 ± 3138 *versus* SWT 131,874 ± 12,328, *P* < 0.0001). This effect could again not be abolished by pre‐treatment with RNase (arbitrary units: SWT+RNase 110,171 ± 14,482, *P* = 0.0005 *versus* CTR), but only with additional proteinase treatment (arbitrary units: SWT+Prot+RNase 877 ± 380.8, *P* = 0.089 *versus* CTR) or sole proteinase treatment (arbitrary units: SWT+Prot 18,713 ± 11,018, *P* = 0.46 *versus* CTR; Fig. 5F) clearly indicating the pivotal role of a RNA/protein complex in SW‐induced TLR3 stimulation.

## Discussion

Heart failure due to ischaemic heart disease represents a global socio‐economic burden 25. Currently, there are no treatment options available for preventing its development after myocardial infarction. SWT represents a promising therapeutic tool for the treatment and regeneration of ischaemic myocardium 5. In the present study, we treated hearts after induction of myocardial infarction and evaluated cardiac function 4 weeks later. Untreated animals developed poor cardiac function with low ejection fraction, impaired contractility and deteriorated systolic and diastolic function. These results could be shown in transthoracic echocardiography and confirmed in haemodynamic pressure–volume measurements. However, functional analyses showed that left ventricular function and myocardial contractility were significantly improved after SWT. In addition, treated animals showed significantly decreased amount of fibrosis of the left ventricle after SWT. These results show clearly a protective effect of SWT concerning ventricular remodelling and cardiac function after myocardial infarction.

Cardiac regeneration after chronic ischaemia has been shown in small and large animal models *via* induction of angiogenesis 5, 12, 26. In addition, cardiac SWT caused improvement of symptoms in patients suffering from chronic ischaemic heart disease. However, we show for the first time that SWT after acute myocardial infarction attenuates the decrease in cardiac function concerning contractility, diastolic and systolic function.

We found increased numbers of capillaries as well as arterioles in treated myocardium. This might be a hint that the protective effect of SWT after myocardial infarction is due to early induction of angiogenesis. The fact that we found increased numbers of arterioles shows that SWT indeed induces the formation of long‐term stable vessels.

Our group recently showed that the SW effect is mediated *via* TLR3 13. However, it remains unclear how the receptor is activated upon mechanical stimulation with SWT. Supernatant from cells pre‐conditioned with SWT showed similar effects on proliferation as the direct application of SWT to cells does. This gave us a strong hint that whatever causes the SWT effect is released into the supernatant. TLR3 is known to be activated by RNA 27. We therefore measured RNA content in the supernatants and could show that it is indeed increased after SWT. However, it remained unclear how the extracellular RNA escapes degradation and passes the cellular membrane to activate intracellular TLR3. The fate of RNA is known to be determined by RNA‐binding proteins 16. We therefore suggested that released RNA binds to a protein and thus passes the cellular membrane. To test this hypothesis, we applied SWT to a tube formation assay in the presence of RNase and proteinase. Interestingly, RNase treatment did not abolish the SWT effect, but only additional treatment with proteinase did indicating the existence of a RNA/protein complex.

In a next step, we aimed to specify which protein is responsible for the observed effects. Gilliet *et al*. have identified LL37 as a key player in the development of psoriasis. Thereby, this antimicrobial peptide gets released upon mechanical stimulation, binds nucleic acids and activates intracellular nucleic acid TLRs 28. We analysed treated cells for LL37 release and LL37 expression and found both increased after SWT. When we added the pre‐marked RNA analogon Poly(I:C) to cells prior to treatment, we found significantly increased uptake of RNA after SWT. These results hint towards involvement of LL37 after SWT. However, it remains unknown whether other proteins/RNA complexes are also involved.

Finally, we analysed whether TLR3 activation after SWT was indeed accomplished by protein/RNA complexes. For this purpose, we performed a TLR3 reporter cell assay. SWT leads to the activation of TLR3, which could not be abolished by RNase treatment, but only with additional proteinase treatment showing that indeed RNA/protein complexes lead to TLR3 activation after SWT.

Summarizing, we could show for the first time that SWT after acute myocardial infarction prevents from left ventricular remodelling and cardiac dysfunction *via* induction of angiogenesis. In addition, we found that the cellular response upon SWT is mediated *via* RNA/protein complexes with involvement of the antimicrobial peptide LL37 activating TLR3. SWT could develop a potent therapeutic tool for the treatment of acute myocardial ischaemia and the prevention of ischaemic heart failure.

## Conflict of interest

None. The authors confirm that there are no conflicts of interest. The sponsors of this study had no role in study design, data collection, analysis and decision to publish or prepare the manuscript.

## Author contributions

C.T. and J.H. designed the experiments and wrote the manuscript. C.T., M.G., U.P. and D.L. performed animal experiments. C.T., L.P. and E.K. performed *in vitro* experiments. C.T., U.P., E.K., E.J.P. and F.N. performed analyses. M.G. revised the manuscript.

## References

[1] Jessup M , Brozena S . Heart failure. N Engl J Med. 2003; 348: 2007–18. 12748317 10.1056/NEJMra021498

[2] Roger VL , Go AS , Lloyd‐Jones DM , *et al* Heart disease and stroke statistics–2012 update: a report from the American Heart Association. Circulation. 2012; 125: e2–220. 22179539 10.1161/CIR.0b013e31823ac046 PMC4440543

[3] McMurray JJ , Adamopoulos S , Anker SD , *et al* ESC Guidelines for the diagnosis and treatment of acute and chronic heart failure 2012: the Task Force for the Diagnosis and Treatment of Acute and Chronic Heart Failure 2012 of the European Society of Cardiology. Developed in collaboration with the Heart Failure Association (HFA) of the ESC. Eur Heart J. 2012; 33: 1787–847. 22611136 10.1093/eurheartj/ehs104

[4] Pearle MS . Shock‐wave lithotripsy for renal calculi. N Engl J Med. 2012; 367: 50–7. 22762318 10.1056/NEJMct1103074

[5] Nishida T , Shimokawa H , Oi K , *et al* Extracorporeal cardiac shock wave therapy markedly ameliorates ischemia‐induced myocardial dysfunction in pigs *in vivo* . Circulation. 2004; 110: 3055–61. 15520304 10.1161/01.CIR.0000148849.51177.97

[6] Aicher A , Heeschen C , Sasaki K , *et al* Low‐energy shock wave for enhancing recruitment of endothelial progenitor cells: a new modality to increase efficacy of cell therapy in chronic hind limb ischemia. Circulation. 2006; 114: 2823–30. 17145991 10.1161/CIRCULATIONAHA.106.628623

[7] Tepekoylu C , Wang FS , Kozaryn R , *et al* Shock wave treatment induces angiogenesis and mobilizes endogenous CD31/CD34‐positive endothelial cells in a hindlimb ischemia model: implications for angiogenesis and vasculogenesis. J Thorac Cardiovasc Surg. 2013; 146: 971–8. 23395097 10.1016/j.jtcvs.2013.01.017

[8] Lobenwein D , Tepekoylu C , Kozaryn R , *et al* Shock wave treatment protects from neuronal degeneration *via* a toll‐like receptor 3 dependent mechanism: implications of a first‐ever causal treatment for ischemic spinal cord injury. J Am Heart Assoc. 2015; 4: e002440. 26508745 10.1161/JAHA.115.002440 PMC4845137

[9] Elster EA , Stojadinovic A , Forsberg J , *et al* Extracorporeal shock wave therapy for nonunion of the tibia. J Orthop Trauma. 2010; 24: 133–41. 20182248 10.1097/BOT.0b013e3181b26470

[10] Schaden W , Thiele R , Kolpl C , *et al* Shock wave therapy for acute and chronic soft tissue wounds: a feasibility study. J Surg Res. 2007; 143: 1–12. 17904157 10.1016/j.jss.2007.01.009

[11] Ottomann C , Stojadinovic A , Lavin PT , *et al* Prospective randomized phase II Trial of accelerated reepithelialization of superficial second‐degree burn wounds using extracorporeal shock wave therapy. Ann Surg. 2012; 255: 23–9. 21775883 10.1097/SLA.0b013e318227b3c0

[12] Fukumoto Y , Ito A , Uwatoku T , *et al* Extracorporeal cardiac shock wave therapy ameliorates myocardial ischemia in patients with severe coronary artery disease. Coron Artery Dis. 2006; 17: 63–70. 16374144 10.1097/00019501-200602000-00011

[13] Holfeld J , Tepekoylu C , Reissig C , *et al* Toll‐like receptor 3 signalling mediates angiogenic response upon shock wave treatment of ischaemic muscle. Cardiovasc Res. 2016; 109: 331–43. 26676850 10.1093/cvr/cvv272

[14] Barton GM , Kagan JC , Medzhitov R . Intracellular localization of Toll‐like receptor 9 prevents recognition of self DNA but facilitates access to viral DNA. Nat Immunol. 2006; 7: 49–56. 16341217 10.1038/ni1280

[15] Ganguly D , Chamilos G , Lande R , *et al* Self‐RNA‐antimicrobial peptide complexes activate human dendritic cells through TLR7 and TLR8. J Exp Med. 2009; 206: 1983–94. 19703986 10.1084/jem.20090480 PMC2737167

[16] Castello A , Fischer B , Eichelbaum K , *et al* Insights into RNA biology from an atlas of mammalian mRNA‐binding proteins. Cell. 2012; 149: 1393–406. 22658674 10.1016/j.cell.2012.04.031

[17] Samarelli AV , Riccitelli E , Bizzozero L , *et al* Neuroligin 1 induces blood vessel maturation by cooperating with the alpha6 integrin. J Biol Chem. 2014; 289: 19466–76. 24860089 10.1074/jbc.M113.530972 PMC4094057

[18] Holfeld J , Tepekoylu C , Kozaryn R , *et al* Shock wave application to cell cultures. J Vis Exp. 2014; 8. doi: 10.3791/51076. 10.3791/51076 PMC4165283 24747842

[19] Primessnig U , Schonleitner P , Holl A , *et al* Novel pathomechanisms of cardiomyocyte dysfunction in a model of heart failure with preserved ejection fraction. Eur J Heart Fail. 2016; 18: 987–97. 27135883 10.1002/ejhf.524

[20] Alesutan I , Voelkl J , Stockigt F , *et al* AMP‐activated protein kinase alpha1 regulates cardiac gap junction protein connexin 43 and electrical remodeling following pressure overload. Cell Physiol Biochem. 2015; 35: 406–18. 25591781 10.1159/000369706

[21] Holfeld J , Tepekoylu C , Blunder S , *et al* Low energy shock wave therapy induces angiogenesis in acute hind‐limb ischemia *via* VEGF receptor 2 phosphorylation. PLoS ONE. 2014; 9: e103982. 25093816 10.1371/journal.pone.0103982 PMC4122398

[22] Holfeld J , Tepekoylu C , Kozaryn R , *et al* Shockwave therapy differentially stimulates endothelial cells: implications on the control of inflammation *via* toll‐Like receptor 3. Inflammation. 2014; 37: 65–70. 23948864 10.1007/s10753-013-9712-1

[23] Albrecht‐Schgoer K , Schgoer W , Holfeld J , *et al* The angiogenic factor secretoneurin induces coronary angiogenesis in a model of myocardial infarction by stimulation of vascular endothelial growth factor signaling in endothelial cells. Circulation. 2012; 126: 2491–501. 23081990 10.1161/CIRCULATIONAHA.111.076950 PMC3839617

[24] Harada M , Qin Y , Takano H , *et al* G‐CSF prevents cardiac remodeling after myocardial infarction by activating the Jak‐Stat pathway in cardiomyocytes. Nat Med. 2005; 11: 305–11. 15723072 10.1038/nm1199

[25] Mozaffarian D , Benjamin EJ , Go AS , *et al* Heart disease and stroke statistics—2015 update: a report from the American Heart Association. Circulation. 2015; 131: e29–322. 25520374 10.1161/CIR.0000000000000152

[26] Holfeld J , Zimpfer D , Albrecht‐Schgoer K , *et al* Epicardial shock‐wave therapy improves ventricular function in a porcine model of ischaemic heart disease. J Tissue Eng Regen Med. 2014; doi: 10.1002/term.1890. [Epub ahead of print]. 10.1002/term.1890 24841341

[27] Akira S , Uematsu S , Takeuchi O . Pathogen recognition and innate immunity. Cell. 2006; 124: 783–801. 16497588 10.1016/j.cell.2006.02.015

[28] Lande R , Gregorio J , Facchinetti V , *et al* Plasmacytoid dendritic cells sense self‐DNA coupled with antimicrobial peptide. Nature. 2007; 449: 564–9. 17873860 10.1038/nature06116

